# Adapting the Dutch Reverse Diabetes2 Now program for Belgian primary care: a quasi-experimental study of effectiveness and transferability

**DOI:** 10.1186/s13690-026-01981-5

**Published:** 2026-06-06

**Authors:** Charlotte Juton, Johan Van der Heyden, Stefanie Vandevijvere

**Affiliations:** 1 Department of Health Information, Food systems and Nutrition, Sciensano, Rue Juliette Wytsman 14, Bruxelles, 1050 Belgique; 2 Department of Health Information, Health Surveys, Sciensano, Rue Juliette Wytsman 14, Bruxelles, 1050 Belgium

## Abstract

**Introduction:**

The prevalence of diabetes in Belgium has steadily increased since 2001, reaching 6.9% in 2024, with type 2 diabetes (T2D) accounting for approximately 90% of cases. Diabetes-related healthcare expenditures were estimated at €2 billion in 2022. The European Care4Diabetes Joint Action aimed to transfer and adapt the evidence-based Dutch lifestyle program Reverse Diabetes2 Now to 12 European countries. This study evaluated the transferability and potential effectiveness of the Care4Diabetes lifestyle intervention on metabolic, behavioral, and subjective health outcomes among Belgian adults with T2D in primary care.

**Research design:**

This quasi-experimental implementation study was conducted in two primary care centers in Wallonia. Forty-three participants initiated the program and 37 completed the 12-month follow-up. The intervention included a 6-month intensive phase with five thematic group sessions and one individual check-up, followed by an additional check-up and a refresher session at Month 12. Primary outcomes were changes in HbA1c and T2D medication use. Secondary outcomes included anthropometric measures, lipid profile, behavioral outcomes, and subjective health indicators. Linear mixed models were used to assess changes over time, accounting for repeated measures.

**Results:**

At Month 12, 46% of participants had no change in T2D medication, 43% underwent medication deintensification, and 11% required intensification. After adjustment for T2D medication changes, HbA1c decreased significantly from baseline to Month 6 by 5.4 mmol/mol (0.49%; *p* = 0.002), but the reduction was attenuated at Month 12 to 2.8 mmol/mol (0.26%; *p* = 0.06). Sensitivity analyses restricted to participants without T2D medication changes showed significant decreases in HbA1c at Month 6 and Month 12. Body weight decreased significantly (− 3.6 kg at Month 12, *p* < 0.001). Improvements were also observed in dietary behaviors and perceived general health, and satisfaction among participants and healthcare providers was high.

**Conclusions:**

The Care4Diabetes program demonstrated good transferability and promising effectiveness in primary care in Wallonia. Larger studies across Belgium are needed to further assess clinical effectiveness and potential economic benefits.

**Supplementary Information:**

The online version contains supplementary material available at 10.1186/s13690-026-01981-5.

**Table:** 

Text box 1. Contributions to the literature
• The rising prevalence and costs of type 2 diabetes highlight the need for effective lifestyle interventions in primary care, but evidence on their implementation and impact remains limited.
• This study demonstrates that a primary care–integrated lifestyle intervention can be implemented in routine practice and may support medication deintensification while maintaining glycemic control and improving patient-reported health.
• These findings support re-centering lifestyle management as the foundation of type 2 diabetes care, alongside appropriate pharmacological treatment, to reduce disease burden.

## Introduction

Diabetes prevalence in Belgium has nearly doubled from 3.4% in 2001 to 6.9% in 2024, with regional disparities (around 6% in Brussels and Flanders vs. 9% in Wallonia) [1]. A 32.8% increase in type 2 diabetes mellitus (T2D) prevalence is projected between 2018 and 2030 [2], with healthcare costs estimated at €2 billion in 2022 [3].

T2D accounts for approximately 90% of all cases [4]. Its main modifiable risk factors include overweight and obesity, unhealthy diet, and physical inactivity. Despite the proliferation of new diabetes therapies, a recent systematic review and meta-analysis of randomized controlled trials has shown that non-pharmacological approaches are more effective in achieving diabetes remission than pharmacological interventions [5]. Diabetes remission is defined by the American Diabetes Association (ADA) as an HbA1c < 48 mmol/mol (6.5%) for at least three months without taking diabetes medication [6]. In line with this evidence, the ADA also emphasizes in its clinical recommendations lifestyle management remains a cornerstone of diabetes care [7]. Despite broad consensus, integrated lifestyle interventions remain underused in primary care due to a stronger focus on pharmacological management and limited experience with multidisciplinary, lifestyle-centered approaches, as well as organizational and resource constraints.

To address these structural and organizational issues, the overarching objective of the Care4Diabetes European Joint Action (C4D JA) was to reduce the burden of T2D and its related risk factors—both at the societal and individual levels—through effective integrated lifestyle-based programs across 12 European countries. The broader expected impacts included improvements in patients’ well-being and quality of life, reductions in diabetes-related healthcare costs, and strengthened health system capacity to adopt innovative, integrated, and sustainable interventions for the prevention and management of T2D.

To achieve these aims, the C4D JA sought to transfer and adapt the evidence-based and reimbursed Dutch lifestyle program “Reverse Diabetes2 Now,” developed by the non-profit organization “Voeding Leeft”. This program has already demonstrated effectiveness in the Netherlands: after two years, 44% of participants reported both reduced medication use and lower HbA1c levels, 42% reported either reduced medication use or lower HbA1c levels, leaving only 14% of participants who reported increased medication use or higher HbA1c levels [8].

The objective of this study was to evaluate the transferability and potential effectiveness of the Care4Diabetes (C4D) lifestyle intervention on metabolic, behavioral, and subjective health outcomes among Belgian adults with T2D in primary care settings.

## Research design and methods

### Study design

This study followed a quasi-experimental implementation trial design. It was conducted in two geographical areas of Wallonia, one of the three Belgian regions: the district of Dinant and the province of Luxembourg. Two primary care centers located in Achêne/Ciney (District of Dinant) and Marche-en-Famenne (Province of Luxembourg) specializing in the management of T2D participated in the study: the Réseau Multidisciplinaire Local de l’Union des Omnipraticiens de l’Arrondissement de Dinant (RML UOAD) and the Maison du Diabète/Chronicare.

### Recruitment and study population

Participants were recruited via primary care centers (websites, social media, mailing lists), direct contact by nurses, and general practitioner referrals. Interested individuals were screened using an eligibility questionnaire.

#### Inclusion criteria

Participants were eligible if they met the following criteria (differences between Group 1 and Group 2 are indicated):

- Age 20–80 years
- Time since T2D diagnosis
  - ◦ Group 1 ≤ 6 years
  - ◦ Group 2 ≤ 10 years
- Currently using medication for T2D
  - ◦ Group 1 excluding SGLT2 inhibitors and GLP-1 receptor agonists
  - ◦ Group 2 including SGLT2 inhibitors or GLP-1 receptor agonists if used ≥ 6 months
- BMI 25–40 kg/m²
- Motivation to engage in lifestyle change
- Ability to participate in the intervention (self-monitoring glycemia, sufficient digital skills, internet access, and availability for group sessions)

The broader criteria in Group 2 were introduced to better reflect current Belgian clinical practice and were agreed upon within the C4D national consortium.

#### Exclusion criteria

Participants were excluded if they presented any of the following conditions:

- Type 1 diabetes or latent autoimmune diabetes in adults
- Use of SGLT2 inhibitors or GLP-1 receptor agonists (Group 1 only)
- Severe COPD
- History of bariatric surgery
- Kidney failure (GFR ≤ 45 ml/min unless approved by a general practitioner)
- Heart failure
- Pregnancy or intention to become pregnant
- Eating disorders or severe psychological disorders
- Vegetarian or vegan diet

### Intervention

#### Providers training

Between September and November 2023, three trainers (a nurse, a dietitian, and the principal investigator) received online training from the Dutch Reverse Diabetes2 Now team. They subsequently delivered a face-to-face French-language train-the-trainers session to 12 healthcare professionals (three nurses, four dietitians, three lifestyle coaches, and two coordinators). One additional coordinator was trained later to replace a staff member on maternity leave.

#### Timeline and program overview

The intervention was delivered in two cohorts:

- Group 1 : January 2024 – December 2024
- Group 2: October 2024 – October 2025

The program comprised two phases:

- Intensive phase: five full-day thematic group sessions delivered over the first 6 months including an individual check-up between thematic day 4 and 5
- Aftercare phase: a refresher session at Month 12 preceded by an individual check-up

Detailed program planning is provided in Supplementary Tables 1, and all program materials are publicly available [9].

### Data collection

Data were collected at baseline, at Month 6 (post–intensive phase), and at Month 12 (aftercare phase).

#### Primary outcomes

- HbA1c
- Change in T2D medication use

#### Secondary metabolic outcomes

- BMI, body weight, waist circumference
- Blood pressure
- Lipid profile (triglycerides, total cholesterol, LDL, HDL)
- Changes in antihypertensive and lipid-lowering medication

#### Behavioral outcomes

- Dietary intake
- Eating habits
- Physical activity
- Self-efficacy

#### Subjective outcomes

- Perceived general health
- Quality of life
- Fatigue
- Sleep problems
- Participant and healthcare provider satisfaction

### Measurement procedures

#### Primary and secondary metabolic outcomes

Fasting blood samples, medication use, anthropometric measures, and blood pressure were collected by trained nurses at baseline, Month 6, and Month 12. All blood analyses were performed by the same accredited laboratories. Medication use was extracted from medical records and verified with participants.

Body weight, BMI, and waist circumference were measured using standardized procedures and calibrated equipment. Blood pressure was measured twice using validated automated devices, and the mean value was recorded.

Changes in T2D medication between baseline and Month 12 were assessed using a two-step procedure capturing both treatment category and dose changes. Treatments were classified hierarchically according to the most intensive therapy prescribed, regardless of whether medications from lower categories were also used:

1. Metformin only
2. DPP-4 inhibitors (gliptins)
3. SGLT2 inhibitors
4. GLP-1 receptor agonists
5. Long-acting insulin
6. Short-acting insulin
7. Other

Transitions to higher categories were defined as intensification, and transitions to lower categories as deintensification. Dose changes within the same category were also considered.

Changes in antihypertensive and lipid-lowering medication were categorized as initiation, discontinuation, or no change between baseline and Month 12.

#### Behavioral outcomes

Behavioral outcomes were assessed using self-reported adapted questionnaires.

Dietary intake was assessed using a tailored version of the Healthy Diet Index food frequency questionnaire [10], developed for use across countries and to address the objectives of the C4D JA. Participants reported the frequency of consumption of major food categories per day or per week.

Eating habits were assessed using a six-item composite score (1–5 Likert scale) reflecting regular eating patterns, enjoyment of food, avoidance of ultra-processed foods, perceived influence on health, confidence in lifestyle change, and conscious health-related choices [11, 12].

Physical activity was assessed across four domains: low-, moderate-, and high-intensity aerobic activities, and muscle-strengthening or mobility activities. Participants reported the average hours per week spent in each [13].

Self-efficacy was measured using an eight-item scale assessing confidence in achieving goals and overcoming challenges [14].

#### Subjective outcomes

Perceived general health was assessed with a five-point self-rating question. Quality of life was measured using the EQ-5D-5 L questionnaire [15].

Fatigue and sleep problems were assessed using single-item questions referring to the past month.

Participant satisfaction with the program was evaluated using Likert-scale items covering program content, structure, perceived benefits, peer support, and use of the Facebook group. Overall satisfaction was assessed with a recommendation score (1–10).

Healthcare professionals’ satisfaction was assessed after the intensive phase (6 months) and after the aftercare phase (12 months). Eleven aspects of the implementation process were evaluated using Likert scales, including clarity of materials, team communication, perceived usefulness, skill development, and likelihood of recommending the program.

### Statistical analyses

Descriptive statistics were reported as percentages for categorical variables and means with standard deviations for continuous variables.

Linear mixed models (LMMs) were used to assess changes across time points. This approach accounts for within-participant correlations in repeated measures and allows inclusion of participants with incomplete observations under the missing-at-random assumption.

Standard LMMs (REML) were used for continuous outcomes, while Poisson or binomial mixed models were applied for count outcomes depending on overdispersion. Models examining HbA1c, weight, BMI, and waist circumference were adjusted for changes in T2D medication. Blood pressure and lipid outcomes were adjusted for changes in antihypertensive and lipid-lowering treatments. Sensitivity analyses were conducted by restricting the sample to participants with no medication changes during follow-up. P-values were adjusted for multiple testing using the Holm method. Statistical significance was set at *p* < 0.05. Analyses were performed using R [16].

### Final sample size

A total of 1,153 individuals were reached through recruitment activities, and 186 were screened for eligibility. Forty-six met the inclusion criteria, and 43 provided written informed consent. Twenty participants were included in Group 1 and 23 in Group 2.

Six participants discontinued the program for reasons unrelated to the intervention (e.g., family illness or loss of motivation), resulting in a final sample of 37 participants. Among the six program sessions, 24 participants (65%) attended all sessions, 12 (32%) attended five sessions, and 1 (3%) attended four sessions.

## Results

### Participant characteristics

Forty-three participants initiated the program, and 37 completed the 12-month follow-up. Participant characteristics are presented in Table 1. Most participants were women (65%), with a mean age of 60.8 years and a mean diabetes duration of 4.0 ± 3.44 years. Most were either employed (38%) or retired (46%). Over half had completed upper secondary or post-secondary education (51%), 35% held a bachelor’s, master’s, or doctoral degree, and 14% had primary or lower secondary education. Overall, 16% had experienced at least one diabetes-related complication.

**Table 1:** Baseline characteristics of study participants

	Overall (*N* = 37)
Gender *N* (%)	
Women	24 (64.9%)
Men	13 (35.1%)
Education *N* (%)	
Primary or secondary inferior	5 (13.5%)
Secondary superior and post-secondary	19 (51.4%)
Bachelor, Master and PhD	13 (35.1%)
Age (years)	
Mean (SD)	60.8 (8.04)
Household income *N* (%)	
Great difficulty to make both ends meet, 1	2 (5.4%)
2	6 (16.2%)
3	14 (37.8%)
4	10 (27.0%)
Can easily make both ends meet, 5	5 (13.5%)
Work situation *N* (%)	
Working	14 (37.8%)
Unemployed	1 (2.7%)
Retired	17 (45.9%)
Housewife/husband	3 (8.1%)
Other	2 (5.4%)
Time since T2D diagnosis (years)	
Mean (SD)	4.00 (3.44)
Diabetes related complications^*^ *N* (%)	
0	31 (83.7%)
1	5 (13.5%)
2	1 (2.7%)

*Diabetes-related complications included angina pectoris, myocardial infarction, stroke, atrial fibrillation, proteinuria, retinopathy, cataract, peripheral neuropathy, and foot ulcer

### Primary outcomes

At Month 12, 46% of participants had no change in T2D medication, 43% underwent deintensification, and 11% intensification; the corresponding proportions at Month 6 were 76%, 22%, and 3%, respectively (Table 2; see Additional file Table 2).

**Table 2:** Changes in T2D, antihypertensive and lipid-lowering medication from baseline to month 12 (*N* = 37)

Changes between baseline and month 12	T2D medication *N* (%)	Antihypertensive medication *N* (%)	Lipid-lowering medication *N* (%)
No change	17 (45.9%)	34 (91.8%)	32 (86.4%)
Deintensification for T2D medication OR Discontinuation for antihypertensive or lipid-lowering medication	16 (43.2%)	2 (5.4%)	1 (2.7%)
Intensification for T2D medication OR Initiation for antihypertensive or lipid-lowering medication	4 (10.8%)	1 (2.7%)	4 (10.8%)

Linear mixed models adjusted for T2D medication changes showed a significant HbA1c reduction of − 5.4 mmol/mol (− 0.49%) from baseline to Month 6 (*p* = 0.002) (Table 3). A smaller reduction was observed at Month 12 (− 2.8 mmol/mol (− 0.26%)), but it was not statistically significant. Sensitivity analyses restricted to participants without medication T2D changes showed significant decreases at both time points (Month 6: −3.6 mmol/mol (− 0.33%), *p* = 0.002; Month 12: −2.2 mmol/mol (− 0.20%), *p* = 0.04).

**Table 3:** Changes in metabolic variables from baseline to Months 6 (T2) and 12 (T3) (*N* = 37)

	Fixed effect	Baseline vs. T2	*P* value^#^	Baseline vs. T3	*P* value
HbA1c^µ^ (mmol/mol)	β = 49.5 SE = 1.60 CI [46.3 ; 52.8]	β = -5.36 SE = 1.60 CI [-8.40; -2.40]	*P* = 0.002	β = -2.80 SE = 1.53 CI [-5.90; 0.11]	*P* = 0.06
HbA1c^µ^ (%)	β = 6.68 SE = 0.15 CI [6.39; 6.98]	β = -0.49 SE = 0.15 CI [-0.77; -0.22]		β = -0.26 SE = 0.14 CI [-0.54; 0.01]	
HbA1c^£^ (mmol/mol)	β = 47.0 SE = 1.09 CI [44.8; 49.2]	β = -3.61 SE = 0.98 CI [-5.68 ; -1.74]	*P* = 0.002	β = -2.19 SE = 0.98 CI [-4.15 ; -0.22]	*P* = 0.04
HbA1c^£^ (%)	β = 6.45 SE = 0.10 CI [6.25; 6.65]	β = -0.33 SE = 0.09 CI [-0.52 ; -0.16]		β = -0.20 SE = 0.09 CI [-0.38 ; -0.02]	
BMI^µ^ (kg/m^2^)	β = 32.6 SE = 1.06 CI [30.5; 34.6]	β = -1.60 SE = 0.26 CI [-2.11; -1.08]	*P* < 0.001	β = -1.28 SE = 0.26 CI [-1.79; -0.77]	*P* < 0.001
BMI^£^ (kg/m^2^)	β = 31.4 SE = 1.09 CI [29.2; 33.5]	β = -1.79 SE = 0.39 CI [-2.57; -1.02]	*P* < 0.001	β = -1.25 SE = 0.38 CI [-2.01; -0.50]	*P* = 0.002
Weight^µ^ (kg)	β = 90.9 SE = 3.68 CI [83.7; 98.2]	β = -4.10 SE = 0.71 CI [-5.49 ; -2.72]	*P* < 0.001	β = -3.59 SE = 0.70 CI [-4.96 ; -2.22]	*P* < 0.001
Weight^£^ (kg)	β = 89.2 SE = 4.37 CI [80.6; 97.7]	β = -4.12 SE = 0.90 CI [-5.89 ; -2.36]	*P* < 0.001	β = -3.52 SE = 0.88 CI [-5.24 ; -1.80]	*P* < 0.001
Waistline^µ*^ (cm)	β = 112 SE = 3.29 CI [106 ; 119]	β = -8.40 SE = 1.24 CI [-10.8; -5.96]	*P* < 0.001	β = -9.20 SE = 1.24 CI [-11.6 ; -6.76]	*P* < 0.001
Waistline^£*^ (cm)	β = 112 SE = 4.8 CI [103; 122]	β = -9.00 SE = 1.70 CI [-12.3; -5.67]	*P* < 0.001	β = -7.44 SE = 1.70 CI [-10.8; -4.11]	*P* < 0.001
Mean systolic^$^ (mmHg)	β = 141 SE = 4.53 CI [132 ; 150]	β = -2.26 SE = 2.99 CI [-8.12 ; 3.59]	*P* = 0.66	β = -2.89 SE = 2.96 CI [-8.70 ; 2.90]	*P* = 0.66
Mean systolic^§^ (mmHg)	β = 140 SE = 3.38 CI [133 ; 146]	β = -2.62 SE = 3.19 CI [-8.87 ; 3.64]	*P* = 0.47	β = -3.81 SE = 3.16 CI [-10 ; 2.39]	*P* = 0.47
Mean diastolic^$^ (mmHg)	β = 86.6 SE = 2.44 CI [81.8 ; 91.4]	β = -1.63 SE = 1.82 CI [-5.20 ; 1.93]	*P* = 0.74	β = -0.17 SE = 1.80 CI [-3.70 ; 3.36]	*P* = 0.93
Mean diastolic^§^ (mmHg)	β = 83.9 SE = 1.77 CI [80.4 ; 87.4]	β =-1.88 SE = 1.92 CI [-5.66; 1.88]	*P* = 0.65	β = -0.10 SE = 1.90 CI [-3.83; 3.69]	*P* = 0.96
Total cholesterol^$^ (mg/dL)	β = 205 SE = 8.22 CI [189 ; 221]	β = -4.42 SE = 4.72 CI [-13.7 ; 4.83]	*P* = 0.66	β = -4.68 SE = 4.77 CI [-14.0 ; 4.65]	*P* = 0.66
Total cholesterol^§^ (mg/dL)	β = 165 SE = 8.55 CI [149 ; 182]	β = -1.72 SE = 4.37 CI [-10.3 ; 6.85]	*P* = 1.00	β = -1.47 SE = 4.37 CI [-10.0 ; 7.10]	*P* = 1.00
LDL^$^ (mg/dL)	β = 122 SE = 7.27 CI [108 ; 137]	β = -4.67 SE = 4.36 CI [-13.2; 3.87]	*P* = 0.57	β = -4.21 SE = 4.40 CI [-12.8; 4.41]	*P* = 0.57
LDL^§^ (mg/dL)	β = 86.4 SE = 7.42 CI [71.9 ; 101]	β = -0.72 SE = 3.74 CI [-8.06 ; 6.63]	*P* = 1.00	β = -0.63 SE = 3.74 CI [-7.97; 6.72]	*P* = 1.00
HDL^$^ (mg/dL)	β = 51.8 SE = 2.96 CI [46.0 ; 57.6]	β = 3.68 SE = 1.17 CI [1.39 ; 5.97]	*P* = 0.004	β = 3.04 SE = 1.18 CI [0.78 ; 5.36]	*P* = 0.01
HDL^§^ (mg/dL)	β = 50.0 SE = 2.68 CI [44.8 ; 52.3]	β = 4.34 SE = 3.06 CI [2.05 ; 6.64]	*P* < 0.001	β = 3.06 SE = 1.17 CI [0.78 ; 5.36]	*P* = 0.01
Triglycerides^$^ (mg/dL)	β = 153 SE = 16.2 CI [121 ; 185]	β = -16.5 SE = 10.1 CI [-36.3 ; 3.26]	*P* = 0.21	β = -11.3 SE = 10.2 CI [-31.3 ; 8.64]	*P* = 0.27
Triglycerides^§^ (mg/dL)	β = 144 SE = 12.6 CI [120 ; 169]	β = -25.9 SE = 9.48 CI [-44.5 ; -7.36]	*P* = 0.02	β = -11.9 SE = 9.48 CI [-30.5 ; 6.60]	*P* = 0.21

µ: Linear mixed-effects models were adjusted for change in T2D medication

£: Sensitivity analyses included participants for whom no change T2D medication was observed (*N* = 17)

*: Owing to measurement errors in Group 1, analyses were restricted to data from Group 2, resulting in a sample size of *N* = 23 for the linear mixed-effects models and *N* = 9 for the sensitivity analyses

$:  Linear mixed-effects models were adjusted for changes in antihypertensive medication for blood pressure outcomes and changes in lipid-lowering medication for lipid outcomes

§: Sensitivity analyses included participants for whom no change in antihypertensive (N=34) or lipid-lowering (N=32) medication was observed, respectively

#: After Holm correction for multiple testing

### Secondary metabolic outcomes

Linear mixed models adjusted for changes in T2D medication showed significant reductions in anthropometric measures (Table 3). Body weight decreased by 4.1 kg at Month 6 and 3.6 kg at Month 12 compared with baseline (both *p* < 0.001), while BMI decreased by 1.6 kg/m² and 1.3 kg/m² at Month 6 and Month 12, respectively (both *p* < 0.001). Similar results were observed in sensitivity analyses restricted to participants without medication changes.

No significant changes were observed in systolic or diastolic blood pressure, total cholesterol, or LDL cholesterol in models adjusted for antihypertensive or lipid-lowering medication, nor in sensitivity analyses (Table 3).

In contrast, HDL cholesterol increased significantly at both time points (+ 3.7 mg/dL at Month 6, *p* = 0.004; +3.0 mg/dL at Month 12, *p* = 0.01), with similar findings in sensitivity analyses.

Triglycerides showed no significant change in the adjusted models. However, sensitivity analyses indicated a significant reduction at Month 6 (− 25.9 mg/dL, *p* = 0.02), which was no longer significant at Month 12 (− 11.9 mg/dL, *p* = 0.21).

### Behavioral outcomes

**Fig. 1:**
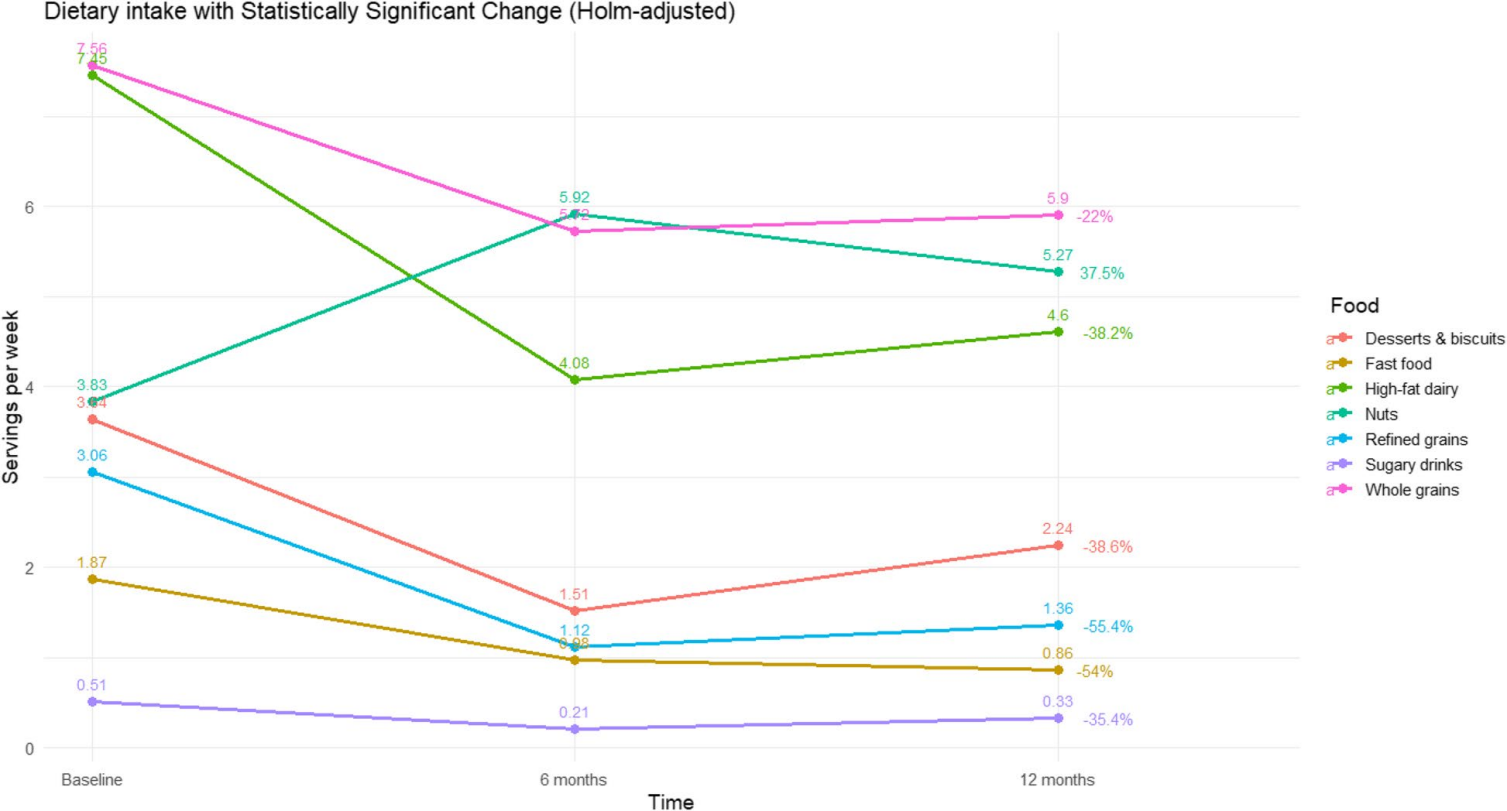
Dietary intake variables with significant changes over time using linear mixed models (*N* = 37)

Significant changes in weekly food consumption were observed for several categories (Fig. 1).

Reductions were found for desserts and biscuits (− 39%), fast food (− 54%), high-fat dairy products (− 38%), refined grains (− 55%), sugary drinks (− 35%), and whole grains (− 22%). In contrast, nut consumption increased (+ 37.5%). Although vegetable (+ 16%) and fruit (+ 11%) intake increased, these changes were not statistically significant (see Additional file Fig. 1).

No significant changes were observed in eating habit scores, physical activity (any intensity), or self-efficacy between baseline and Month 12 (Table 4).

**Table 4:** Changes in behavioral and subjective variables from baseline to Months 6 and 12 (*N* = 37)

	Fixed effect	Baseline vs. T2	*P* value^#^	Baseline vs. T3	*P* value
Behavioral outcomes					
Eating habits (1 to 5 units)	β = 3.97 SE = 0.10 CI [3.77 ; 4.17]	β = 0.27 SE = 0.09 CI [0.07 ; 0.44]	*P* = 0.09	β = 0.09 SE = 0.09 CI [-0.09 ; 0.27]	*P* = 1.00
Self-efficacy score (1 to 5 units)	β = 3.79 SE = 0.12 CI [3.55 ; 4.03]	β = -0.05 SE = 0.11 CI [-0.27 ; 0.15]	*P* = 1.00	β = -0.13 SE = 0.11 CI [-0.33 ; 0.08]	*P* = 1.00
Low physical activity (hours/week)	β = 5.55 SE = 1.40 CI [2.79 ; 8.31]	β = 1.44 SE = 1.62 CI [-1.74 ; 4.62]	*P* = 1.00	β = 0.32 SE = 1.62 CI [-2.86 ; 3.49]	*P* = 1.00
Moderate physical activity (hours/week)	β = 2.30 SE = 0.75 CI [0.82 ; 3.78]	β = 0.63 SE = 0.69 CI [-0.73 ; 1.99]	*P* = 1.00	β = 1.37 SE = 0.69 CI [0.01 ; 2.73]	*P* = 0.58
High physical activity (hours/week)	β = 0.21 SE = 0.27 CI [-0.32 ; 0.74]	β = 0.65 SE = 0.36 CI [-0.05 ; 1.36]	*P* = 0.66	β = 0.71 SE = 0.36 CI [0.001 ; 1.41]	*P* = 0.58
Muscle strengthening (hours/week)	β = 2.45 SE = 0.97 CI [0.55 ; 4.36]	β = -1.15 SE = 1.37 CI [-3.84 ; 1.53]	*P* = 1.00	β = -0.77 SE = 1.37 CI [-3.46 ; 1.91]	*P* = 1.00
Subjective outcomes					
Perceived general health (1 to 5 units)	β = 2.95 SE = 0.13 CI [2.68 ; 3.21]	β = 0.32 SE = 0.16 CI [0.01 ; 0.64]	*P* = 0.27	β = 0.57 SE = 0.16 CI [0.25 ; 0.88]	*P* = 0.01
Quality of life Eq. 5D5L (-0.532 to 1 unit)	β = 0.70 SE = 0.04 CI [0.62 ; 0.78]	β = 0.03 SE = 0.02 CI [-0.02 ; 0.07]	*P* = 1.00	β = 0.01 SE = 0.02 CI [-0.04 ; 0.05]	*P* = 1.00
Fatigue (1 to 5 unit)	β = 3.43 SE = 0.19 CI [3.06 ; 3.80]	β = -0.51 SE = 0.21 CI [-0.93 ; -0.10]	*P* = 0.12	β = -0.22 SE = 0.21 CI [-0.63 ; 0.20]	*P* = 1.00
Sleep problems (1 to 5 units)	β = 3.70 SE = 0.20 CI [3.30 ; 4.11]	β = -0.27 SE = 0.24 CI [-0.74 ; 0.20]	*P* = 1.00	β = -0.46 SE = 0.24 CI [-0.93 ; 0.01]	*P* = 0.29

#: After Holm correction for multiple testing

### Subjective outcomes

Several subjective outcomes were assessed during the study period (Table 4). Participants reported a significant improvement in perceived general health between baseline and Month 12 (β = 0.57, SE = 0.16, 95% CI [0.25; 0.88], *p* = 0.01), whereas overall quality of life did not change significantly. Non-significant reductions were observed for sleep problems and fatigue.

Participant and healthcare professional satisfaction were high, with mean scores ranging from 4.0 to 4.8 and 3.5 to 5.0, respectively, on a 5-point scale (see Additional file Figs. 2 and 4, and 5). Program recommendation scores were also high (see Additional file Figs. 3 and 6).

## Discussion

### Comparison between C4D and other lifestyle interventions in primary care settings

Robust evidence on lifestyle interventions for T2D implemented in primary care remains limited [17–19]. Nevertheless, integrated lifestyle programs have shown improvements in glycemic control and weight loss and, in some cases, diabetes remission (HbA1c < 48 mmol/mol (6.5%) for at least two months without medication) [18, 19]. Most studies, however, focus mainly on nutrition and/or physical activity, often without addressing sleep or stress, and detailed intervention descriptions are frequently lacking, limiting comparisons of content and intensity.

Several interventions include meal-replacement strategies [18, 19], which may hinder large-scale implementation due to limited acceptability and long-term adherence [20, 21]. In contrast, culturally adapted dietary approaches such as the one implemented in this study may be more acceptable and sustainable. Another small study delivered a combined nutrition and physical activity intervention but included only minimal nutritional guidance (e.g., vinegar diluted in water or added to salads). Regarding intervention intensity, one study reported 25–37.5 h of participant contact compared with approximately 42 h in the present program [18].

Overall, comparisons across lifestyle interventions remain challenging due to heterogeneity in program components (e.g., nutrition, physical activity, stress, sleep) and intensity. More standardized reporting frameworks and transparent descriptions of intervention content would improve comparability and facilitate evidence synthesis.

### Comparison between C4D and the same program Reverse Diabetes2 Now in the Netherlands

The C4D program was adapted from the Dutch Reverse Diabetes2 Now (RD2) intervention developed by the non-profit organization Voeding Leeft. The Belgian version closely mirrored the original program in content and structure, with only minor cultural adaptations to dietary recipes, allowing meaningful comparison between the two interventions.

In the original RD2 publication (2019) [22], which evaluated outcomes between baseline and Month 6, the mean reduction in HbA1c was similar to that observed in the present study (− 5.5 mmol/mol (-0.5%) in both programs; Supplementary Table 2), despite higher baseline HbA1c levels in the Dutch cohort. This suggests that the Belgian adaptation achieved comparable glycemic improvements in a population with a slightly more favorable metabolic profile at baseline.

Medication deintensification occurred earlier in the RD2 program than in the Belgian implementation. This difference may partly reflect baseline treatment patterns: approximately 73% of RD2 participants used sulfonylureas and/or insulin with metformin, therapies often discontinued when carbohydrate intake is reduced to avoid hypoglycemia. In contrast, C4D participants were more frequently treated with metformin alone or with GLP-1 receptor agonists, which are not associated with hypoglycemia risk and therefore do not systematically require medication reduction.

Differences may also reflect clinical decision-making practices. While medication reduction was pursued earlier in the Dutch cohort, the Belgian approach prioritized glycemic stability and cautious follow-up. Nevertheless, by Month 12, 48.6% of C4D participants had reduced their medication while maintaining a mean HbA1c of 48 mmol/mol (6.5%). In contrast, 24-month follow-up data from RD2 showed no significant change in HbA1c compared with baseline (56.3 (7.5%) vs. 58.5 (7.0%) mmol/mol), although 67% of participants used less medication, suggesting a stronger emphasis on medication reduction in the Dutch context [23].

Anthropometric improvements were comparable across programs. Mean weight loss (− 4.3 kg in C4D vs. −4.9 kg in RD2) and BMI reductions (− 1.4 vs. −1.7 kg/m²) were of similar magnitude, although Belgian participants had slightly higher baseline BMI (32.6 vs. 31.2 kg/m²). In RD2, around 31% of participants were treated with metformin, sulfonylureas, and insulin at baseline, and approximately half discontinued insulin during the intervention. Because insulin therapy is associated with weight gain [24], its discontinuation may partly explain the slightly greater weight loss observed in the RD2 program.

Larger reductions in total cholesterol, LDL cholesterol, and triglycerides were reported in RD2; however, baseline lipid levels were higher in the Dutch cohort, which likely contributed to the greater absolute changes. Participants in C4D already presented more favorable lipid profiles at baseline, with LDL cholesterol and triglycerides below commonly recommended thresholds (< 100 mg/dL and < 150 mg/dL, respectively) and HDL cholesterol close to the target value (> 50 mg/dL) recommended by the American Diabetes Association [25]. Moreover, changes in lipid-lowering medication were not considered in the RD2 analysis, which may have influenced these results.

Improvements in perceived general health were reported in both programs.

### Transferability, acceptability and scalability

Satisfaction among both participants and healthcare providers was high, highlighting the acceptability and transferability of the C4D program when culturally adapted. These findings support the feasibility of implementing structured lifestyle interventions across healthcare systems. In Belgium, however, an established federal care pathway for people with T2D already exists; C4D should therefore be positioned as a complementary intervention rather than an overlapping service.

A key strength of C4D is its group-based format, whereas routine diabetes care is primarily delivered through individual consultations due to the organizational resources required for group programs. As primary care increasingly shifts toward integrated care models in response to the growing burden of chronic diseases [26, 27], including T2D, group-based lifestyle interventions may become more relevant.

Scalability is further supported by the train-the-trainers model, which enables trained professionals to disseminate knowledge and skills to additional providers. Improvements in providers’ lifestyle-related knowledge and skills between the two implementation rounds suggest a learning-curve effect. Training a larger number of healthcare professionals (12 instead of 8) also strengthens the program’s capacity for future scale-up and sustainable implementation.

### Sustainability and long term support needs

Newer pharmacological treatments, such as GLP-1 receptor agonists and SGLT2 inhibitors, are increasingly used in T2D care; however, lifestyle modification remains a cornerstone of disease management and cannot be replaced by pharmacotherapy alone. Lifestyle-based interventions can complement medical treatment, helping prevent disease progression and treatment intensification [28], and in some cases enabling medication deintensification or remission [29, 30].

In this study, a greater proportion of participants reported medication deintensification between Month 6 and Month 12. However, sustaining behavioral change appears to require ongoing support. Participants expressed a need for additional sessions, reflected in lower satisfaction during the aftercare phase, which included only one session compared with five during the intensive phase. These findings highlight the importance of structured follow-up and refresher components to maintain benefits and prevent relapse [31, 32].

### Public health and economic implications

Progression of T2D is associated with important clinical and economic consequences, including disease worsening, treatment intensification, reduced quality of life, higher complication and hospitalization rates, productivity loss, absenteeism, and increased healthcare costs [28, 33]. Given that T2D is a major contributor to medication-related healthcare expenditure in Belgium [3], interventions such as C4D —which enable medication deintensification while maintaining HbA1c levels below 48 mmol/mol (6.5%) and improving perceived general health—may help reduce both disease burden and long-term healthcare costs.

### Strength and limitations

A key strength of this study is its quasi-experimental design, which is well suited to real-world primary care settings and provides strong external validity. The design also reflects real-world patient engagement and attrition patterns, increasing the relevance of the findings for routine practice. Another strength is the inclusion of medication change as a primary outcome, offering a clinically meaningful perspective beyond glycemic control. In addition, linear mixed models appropriately accounted for repeated measures and missing-at-random observations while adjusting for changes in T2D, lipid-lowering, or antihypertensive medications.

However, several limitations should be noted. First, the absence of an a priori power calculation and the relatively small sample size may have limited statistical power. Second, the specific geographical setting may restrict generalizability. Finally, although participants with primary education were included, the sample size did not allow subgroup analyses by educational level, limiting insights into potential equity-related differences.

### Implications for future research

The C4D program showed good transferability in Belgian primary care and was associated with improvements in T2D management. The evidence generated by this study could be strengthened through a pragmatic randomized controlled trial comparing routine care with the C4D program. Conducting such a study in a larger and more representative Belgian sample would allow a more robust evaluation of clinical effectiveness and economic benefits. Additionally, assessing the program at the prediabetes stage could be particularly valuable, as early prevention of T2D substantially reduces all-cause mortality [34] and may provide even greater benefits for patients and healthcare systems.

## Supplementary Information

Supplementary Material 1.

## Data Availability

The datasets generated during and/or analysed during the current study are available from the corresponding author on reasonable request.
