# Supplementary material for: Adapting the Dutch Reverse Diabetes2 Now program for Belgian primary care: a quasi-experimental study of effectiveness and transferability
Source: Arch Public Health. 2026 Jun 6;84:130. doi: 10.1186/s13690-026-01981-5 (PMC13262315; doi:10.1186/s13690-026-01981-5)
Supplement: Supplementary file 1 — Supplementary Material 1. [file 13690_2026_1981_MOESM1_ESM.docx]

Supplementary table 1. Comprehensive description of the Care4Diabetes program curriculum.

| J1 | 1. **Welcome and Introduction**  - Team and participants presentation  1. **Understanding Diabetes**  - Differences between Type 1 and Type 2 Diabetes - Physiology of Type 2 Diabetes - Introduction to Insulin Resistance as the Main Underlying Issue  1. **Diagnosis of Type 2 Diabetes**  - Round table   **Quick Physical activity break**  **Lunch Break**   1. **Nutrition and Type 2 Diabetes**  - Role of nutrition in insulin resistance - Strategies to optimize the management of Type 2 Diabetes - Nutritional recommendations of C4D  1. **Self-Monitoring and Patient Empowerment**  - Importance of self-monitoring of blood glucose  1. **Workshop Conclusion**  - Reflection: *What do you take away from this day?* - Group discussion and feedback |
| --- | --- |
| J2 | 1. **Fasting Blood Glucose Measurement**  - Measurement and interpretation - Explanation of results   Breakfast   1. **Review of C4D Nutritional Recommendations**  - Key reminders and practical tips  1. **Behavioral and Social Support**  - What motivates you to eat healthy? - How can relatives and family members provide support?  1. **Practical Cooking Workshop**  - Preparing healthy and delicious recipes - Making your own balanced meals  1. **Understanding Food Choices**  - How to read and understand food labels  1. **Post-Meal Self-Monitoring**  - Blood glucose measurement 2 hours after lunch - Interpretation and discussion  1. **Community Support**  - Presentation of the group Facebook page and its purpose  1. **Workshop Conclusion**  - Reflection: *What do you take away from this day?* - Group discussion and feedback |
| J3 | 1. **Welcome and Participant Check-In**  - Feedback on nutritional changes - Discussion on motivation, support from relatives, barriers, and facilitating factors  1. **Physical Activity and Type 2 Diabetes**  - Benefits of physical activity - Importance of regular exercise in Type 2 Diabetes management  1. **Adapted Physical Activity Session** *(if feasible) /* **Physical activity break (walking)** 2. **Care4Diabetes Physical Activity Recommendations**  - Key principles and safety considerations - How to integrate physical activity into daily life  1. **Local Resources and Opportunities**  - Overview of available regional programs, facilities, and support networks  1. **Participant Engagement Discussion**  - What is your favorite type of physical activity or sport? - What motivates you most in the proposed activities?  1. **Action Planning**  - What concrete actions are you going to implement after this workshop?  1. **Step Count Group Challenge** |
| J4 | 1. **Feedback on Physical Activity Changes**  - Sharing experiences since the last session - Discussion on motivation, support from relatives, barriers, and facilitating factors  1. **Relaxation Activity *(if feasible)***  - Guided relaxation session (e.g., Tibetan bowls or similar technique)  1. **Stress and Type 2 Diabetes**  - Theoretical overview of stress - Understanding the relationship between stress and blood glucose regulation   **Lunch Break – Potluck Meal**   1. **Mindful Eating and Food Awareness**  - Food tasting with focus on food consciousness and sensory experience  1. **Understanding Personal Stress Patterns**  - What are your personal stress-related characteristics? - What signals indicate that you are stressed? - What strategies or solutions do you use to cope with stress?  1. **Physical Activity Break** 2. **Workshop Reflection and Closing**  - Reflection: *What do you take away from this day?* - Group discussion and participant feedback |
| J5 | 1. **Feedback on Relaxation Practices**  - Sharing experiences since the last session - Discussion on motivation, barriers, and facilitating factors  1. **Sleep and Health**  - Theoretical overview of sleep and its role in overall health - Understanding the relationship between sleep and Type 2 Diabetes  1. **Improving Sleep Quality**  - Practical strategies to enhance sleep habits - Sleep hygiene recommendations and daily routine adjustments  1. **Healthy Barbecue Session**  - Preparing balanced and healthy barbecue options - Practical tips for healthier food choices during social gatherings - Group meal and social exchange |
| J6 | The refresher day aims to review the key recommendations and concepts covered throughout the program. It provides participants with an opportunity to:   - Reinforce knowledge and skills acquired during previous sessions - Clarify remaining questions or uncertainties - Receive additional information and support based on individual needs - Share experiences and challenges encountered since completing the program - Develop or adjust a personalized action plan to support long-term lifestyle changes |

Supplementary table 2. Comparison of clinical outcomes between the Belgian and Dutch programs from baseline to Month 6.

| Change in type 2 diabetes medication between baseline and month 6 | Care4Diabetes (Belgium)  N=37  N (%) | Reverse Diabetes 2 (Netherlands)  N=72  N (%) |
| --- | --- | --- |
| No change | 28 (75.7%) | 36 (50.0%) |
| Deintensification | 8 (21.6%) | 35 (48.6%) |
| Intensification | 1 (2.7%) | 1 (1.4%) |

Supplementary table 3. Comparison of change in type 2 diabetes medication between the Belgian and Dutch programs from baseline to Month 6.

|  | Care4Diabetes (Belgium)  N=37 | | | | Reverse Diabetes 2 (Netherlands)^µ^  N=72 | | | |
| --- | --- | --- | --- | --- | --- | --- | --- | --- |
|  | N | Baseline  Mean (SD) | Month 6  Mean (SD) | Mean difference (baseline - Month 6) | N | Baseline  Mean (SD) | Month 6  Mean (SD) | Mean difference  (baseline - Month 6) |
| HbA1c (mmol/mol) | 37 | 51 (11) | 45 (6.7) | -5.5 | 72 | 59 (12) | 53 (13) | -5.5 |
| HbA1c (%) |  | 6.77 (0.99) | 6.3 (0.6) | -0.5 |  | 7.5 (1.1) | 7.0 (1.2) | -0.5 |
| BMI (kg/m^2^) | 37 | 32.6 (4.7) | 31.2 (4.6) | -1.4 | 63 | 31.2 (4.2) | 29.5 (4.5) | -1.7 |
| Weight (kg) | 37 | 92.6 (16.6) | 88.3 (16.6) | -4.3 | 65 | 93.2 (14.3) | 88.3 (14.9) | -4.9 |
| Total cholesterol (mg/dl) | 37 | 170 (51.3) | 164 (43) | -6 | 34 | 190 (46.4) | 155 (65.7) | -35 |
| LDL (mg/dl) | 37 | 91.9 (45.0) | 85.9 (38.2) | -6 | 41 | 116 (42.5) | 112 (46.4) | -15.5 |
| HDL (mg/dl) | 37 | 49.7 (13.3) | 53.3 (16.0) | +3.6 | 41 | 46.4 (11.6) | 46.4 (11.6) | 0 |
| Triglycerides (mg/dl) | 37 | 143 (56.7) | 126 (80.8) | -17 | 38 | 168 (79.7) | 133 (62) | -35.4 |

µ: results are taken from Pot et al. 2019

Supplementary Figure 1. Dietary intake variables exhibiting non statistically significant changes over time as estimated by linear mixed models, with continuous outcomes analyzed using REML and count outcomes using Poisson or binomial mixed models.


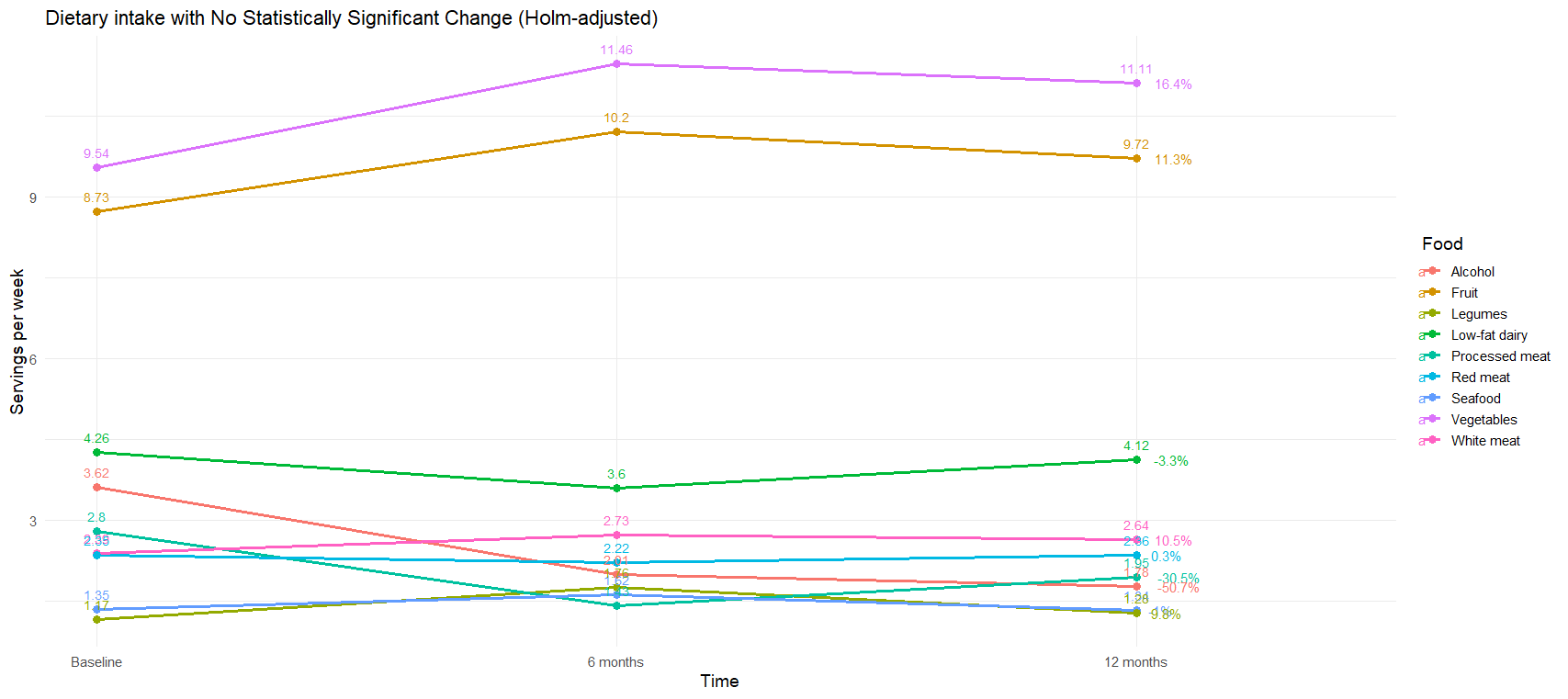


Supplementary Figure 2. Participants satisfaction regarding several characteristics of the Care4Diabetes program.

Supplementary Figure 3. Distribution of participants’ likelihood of recommending the Care4Diabetes program, rated from 1 (least likely) to 10 (most likely).


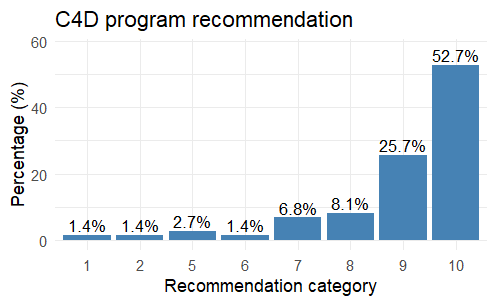


Supplementary Figure 4. Healthcare professionals satisfaction regarding several characteristics of the Care4Diabetes program after the intensive phase in each round.

Supplementary Figure 5. Healthcare professionals satisfaction regarding several characteristics of the Care4Diabetes program after the aftercare phase in each round.

Supplementary Figure 6. Likelihood of recommending the program Care4Diabetes to other healthcare professionals.
